# Walk-and-talk versus conventional psychotherapy for men with depressive symptoms: study protocol of a randomised controlled trial

**DOI:** 10.1136/bmjopen-2026-122564

**Published:** 2026-08-26

**Authors:** Casey Phyllis Regan, Andrea Dickmeyer, Robert O’Hara, Sean Anthony Halpin, Jordan James Smith, Philip Morgan, Ryan J Drew, Victoria McCreanor, Sarah Ruth Valkenborghs, Elliot Waters, Zac Seidler, Myles D Young

**Affiliations:** 1 School of Science, The University of Newcastle, Callaghan, New South Wales, Australia; 2 Global Sport and Movement Collaborative, The University of Newcastle, Callaghan, New South Wales, Australia; 3 Hunter Medical Research Institute, New Lambton Heights, New South Wales, Australia; 4 School of Education, The University of Newcastle, Callaghan, New South Wales, Australia; 5 School of Biomedical Sciences and Pharmacy, The University of Newcastle, Callaghan, New South Wales, Australia; 6 School of Medicine and Public Health, The University of Newcastle, Callaghan, New South Wales, Australia; 7 AusHSI, Centre for Healthcare Transformation, Queensland University of Technology, Brisbane, Queensland, Australia; 8 Centre for Youth Mental Health, The University of Melbourne, Melbourne, Victoria, Australia; 9 Orygen, Parkville, Victoria, Australia; 10 Movember Institute of Men’s Health, Melbourne, Victoria, Australia

**Keywords:** MENTAL HEALTH, Exercise, Depression & mood disorders, Randomized Controlled Trial, Psychological Stress, Psychosocial Intervention

## Abstract

**Introduction:**

Walk-and-talk therapy integrates psychological support and physical activity conducted in outdoor environments, and has potential to better align with men’s preferences and masculine norms around emotional communication. Conventional indoor psychotherapy is effective for treating depression but can be less engaging for men, leading to less satisfaction and high rates of dropout. This trial aims to determine whether walk-and-talk therapy offers men with depressive symptoms clinically meaningful benefits over conventional indoor therapy.

**Methods and analysis:**

This assessor-blinded, parallel group randomised controlled trial is comparing the efficacy of walk-and-talk therapy to conventional indoor therapy for men with depressive symptoms. After randomisation, all participants receive 10 fortnightly 1-hour individual psychotherapy sessions over 20 weeks as either: (1) conventional indoor therapy (sitting down indoors) or (2) walk-and-talk therapy (walking outdoors). Participants are assessed at ‘pre-intervention’ (baseline), ‘post-intervention’ (5 months post baseline) and ‘follow-up’ (12 months post baseline) time points. The primary outcome is change in overall psychological distress (21-item version of the Depression, Anxiety and Stress Scale (DASS-21)) at post-intervention. Secondary outcomes include male-type depression symptoms, mental well-being, suicidal ideation and quality of life. Costing data is being collected for cost-effectiveness analysis. Linear mixed models will be used to examine the impact of group (conventional indoor vs walk-and-talk), time (baseline, 5-month and 12-month) and the group-by-time interaction for primary and secondary outcomes.

**Ethics and dissemination:**

This study is approved by the University of Newcastle Human Research Ethics Committee (H-2024-0090). Results will be published in an open access peer-reviewed journal and disseminated through conference presentations.

**Trial registration number:**

ACTRN12624000794505.

**STRENGTHS AND LIMITATIONS OF THIS STUDY**

- Randomised controlled design with an active comparator (conventional indoor therapy).
- Preregistered analysis plan using intention-to-treat principles and 12-month follow-up assessment.
- Broad range of psychological, behavioural, social and economic outcomes with advanced analyses (eg, mediation/moderation).
- Reliance on psychologist self-report to assess treatment adherence may introduce bias in fidelity measurement.
- Self-selected, university-based sample may limit generalisability to routine clinical and broader health service populations.

## Introduction

 In Australia and internationally, the burden of depressive disorders in men is growing.^1–3^ Between 1990 and 2021, both the total number of men with depression and the years of healthy life lost to depression have almost doubled globally.^3^ Current estimates suggest one in eight men will now experience depression over the lifespan.^4^ These data are also reflected in Australia, where depression is one of the most common mental health disorders in men.^5^

Despite recent increases in men’s help-seeking,^6^ men remain less likely to seek psychological support than women.^7–9^ For example, in Australia, 51% of women experiencing psychological distress consulted a health professional, compared with 37% of men.^5^ In a report from the world’s largest longitudinal men’s health study,^10^ only one-quarter of men indicated that they were likely to seek help from a mental health professional if they experienced an emotional or personal problem.^11^ This help-seeking gap may in part explain why women are twice as likely to be diagnosed with depression, yet men are three times more likely to die by suicide.^12–14^

These data suggest that current mental health treatment options are not engaging large numbers of men who need help. For example, while psychotherapy is effective for treating depression,^15^ estimates suggest up to 45% of men drop out of therapy prematurely.^16^ One contributing factor is that conventional indoor therapy settings may not align with men’s preferences or social conditioning. The dominant cultural narrative around masculinity in many western countries, including Australia, rewards emotional stoicism and self-reliance,^17 18^ making it challenging for many men to express vulnerability in the formal, face-to-face structure of conventional therapy. This discomfort is a key reason cited for discontinuing therapy, with many men reporting that therapy ‘didn’t feel right’.^16^ While this may reflect the complexity of engaging and assisting men with mood disorders, it reinforces the need for gender-specific treatments that men with depression find appealing.^19 20^

Internationally, the groundswell of support to improve men’s therapy outcomes is growing. In 2018, the American Psychological Association released their first set of guidelines for working with men and boys, compelling psychologists to provide gender sensitive care by ‘changing the structure of interventions to be more congruent for the male client’.^21^ Researchers are also proposing frameworks to tailor men’s health programmes for improved accessibility and engagement.^22 23^ Despite this, there is little experimental evidence to highlight which treatment modifications are most important for improving men’s outcomes in therapy. Recent reviews of the field have highlighted a significant under-representation of men in psychotherapy trials targeting depression,^24 25^ with only 3% of studies focused on men in the past 50 years.^25^ To address this knowledge gap and inform actionable evidence-based strategies for clinical practice, rigorous trials targeting men are needed.

### Walk-and-talk therapy

An alternate mode of psychotherapy that has potential to engage men is walk-and-talk therapy.^26^ Walk-and-talk therapy refers to usual psychotherapeutic discussions conducted by a licensed mental health therapist while walking alongside a client on a predefined path outdoors, often using nature as the backdrop.^27^ Compared with the conventional indoor therapy mode, walk-and-talk therapy naturally requires less direct eye contact due to the shoulder-to-shoulder orientation,^28^ allowing for shared ownership of the physical space where therapy takes place. Outdoor therapy sessions may de-emphasise the perceived formality and inherent power imbalance between clients and therapists,^28^ which can help men feel less pressure and more comfortable when discussing sensitive topics.^29^ Walk-and-talk therapy may provide mental health benefits from the physical activity of walking^30^ and/or from exposure to nature when sessions are conducted in a natural setting, given humans’ innate attraction to nature.^31^

While experimental data are lacking, a meta-synthesis of 38 qualitative studies found that outdoor therapy was a highly beneficial and valued practice by both service end-users (n=163) and clinicians (n=322).^28^ Although some barriers were reported, such as privacy concerns and poor weather, clinicians generally found these issues could be managed with adequate prescreening and planning, even in higher risk in-patient groups.^28^ Further, a Canadian pilot trial found that walking psychotherapy was both feasible and acceptable for patients in an outpatient trauma therapy programme to reduce sedentary behaviour.^32^ These insights suggest walk-and-talk therapy is supported by expert clinical opinion and end-user preferences, which are two of the three cornerstones of evidence-based psychotherapy. Yet, to date no clinical trials have investigated the comparative effectiveness of walk-and-talk versus conventional indoor therapy in men or otherwise.^28^ Without high-quality clinical trial data confirming effectiveness, safety, and cost-effectiveness of walk-and-talk therapy, it is less likely that this practice will be integrated into standard care.

To address this evidence-gap, we recently conducted a preregistered pilot study comparing walk-and-talk therapy with conventional indoor therapy for men experiencing depressive symptoms.^33^ At the conclusion of the study, all feasibility benchmarks were achieved (eg, recruitment capability, attendance, retention), and while reductions in depression were similar across groups, men in the walk-and-talk condition reported greater improvements in anxiety, stress and overall psychological distress. These findings suggest that taking therapy outdoors may offer broad mental health benefits while being similarly beneficial for depression. While promising, these preliminary findings need to be confirmed through a sufficiently powered clinical trial.

Building on this, the aim of this trial is to test the comparative effectiveness of walk-and-talk therapy on men’s overall psychological distress. We expect this trial will determine whether this approach provides clinically meaningful advantages over conventional indoor therapy for men with depressive symptoms. We hypothesise that walk-and-talk therapy will produce greater reductions in overall psychological distress than conventional indoor therapy.

## Methods

### Study design

The study design is a parallel-group superiority randomised trial repeated across three cohorts. In each cohort, participants are assessed at baseline, 5 months (post-intervention) and 12 months (follow-up). After completing baseline measures, participants were randomly allocated to either conventional indoor therapy (sitting down indoors) or walk-and-talk therapy (walking outdoors). At the time of writing, recruitment, intervention delivery and post-intervention assessments have been completed across all three cohorts, with 12-month follow-up assessments currently underway. The study was approved by the University of Newcastle Human Research Ethics Committee (HREC) (H-2024-0090) and was prospectively registered with the Australian New Zealand Clinical Trials Registry (ACTRN12624000794505, ANZCTR - Registration, 27 June 2024). Any modifications to the study protocol would be submitted to HREC for approval prior to implementation and updated in the publicly available trial registry. No substantial protocol amendments have been made up to this point. All participants receive $A10 vouchers for completing the post-intervention survey at 5 months and follow-up survey at 12 months ($A20 in total). The reporting of this study will adhere to the Consolidated Standards of Reporting Trials (CONSORT) guidelines.^34^

#### Participants

**Figure 1:**
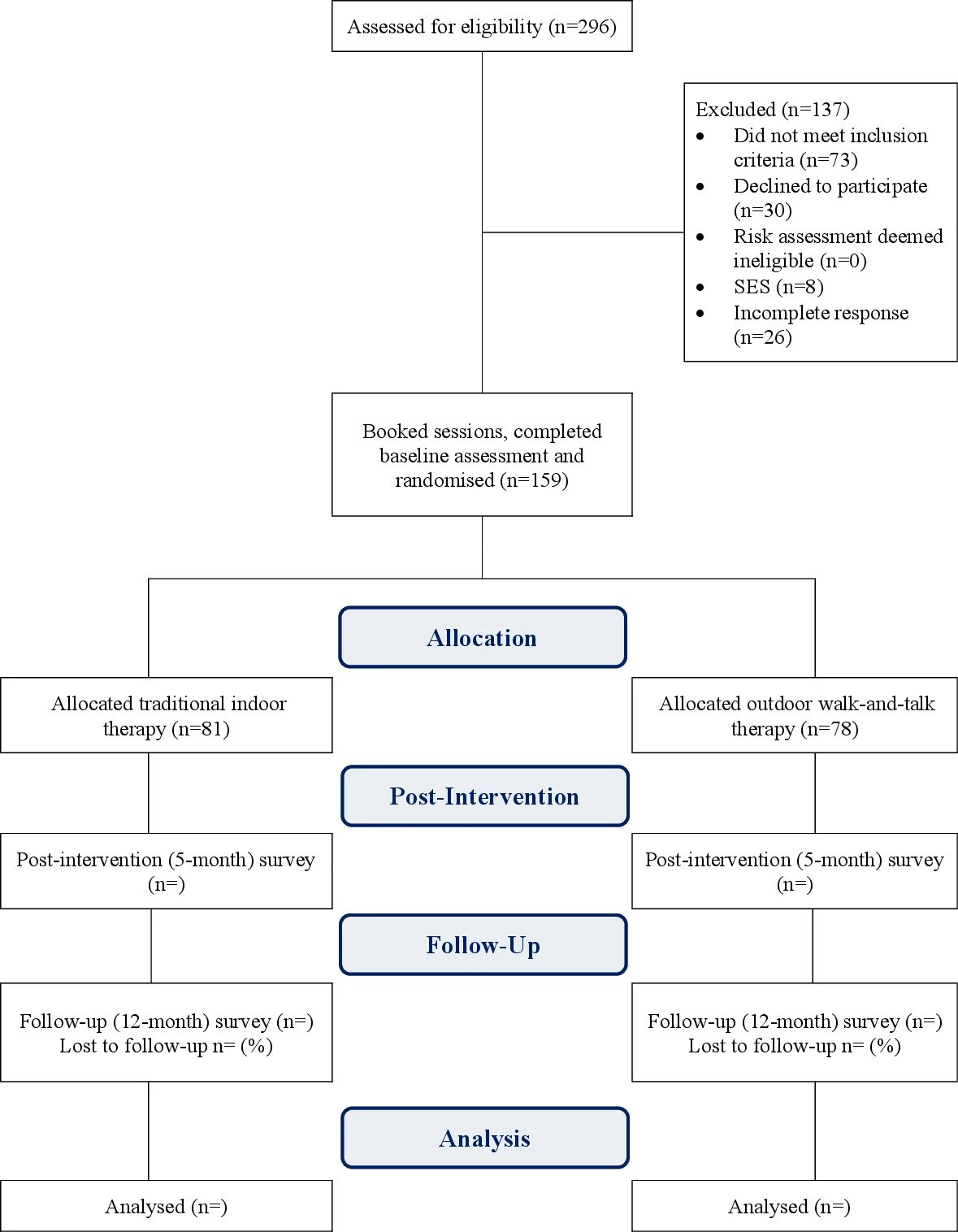
CONSORT flowchart. CONSORT, Consolidated Standards of Reporting Trials; SES, socio-economic status.

Participants were recruited from the Hunter region of New South Wales (NSW), Australia, from June to July 2024 (cohort 1), January to February 2025 (cohort 2) and June to July 2025 (cohort 3). Figure 1 displays the study flow-chart for the trial.

Recruitment strategies included paid social media advertising (eg, Facebook), flyers distributed across a range of community venues (eg, shops, libraries, barbers) and the Callaghan University campus, emails to a registry of men who were interested in hearing about mental health research opportunities, and local media exposure (eg, radio interview). For equity purposes, we set an a priori recruitment equity target of at least 30% of participants from areas of low socio-economic areas, determined by cross-referencing residential postcode at recruitment with the Index of Relative Socio-Economic Advantage and Disadvantage database.^35^ Each postcode was assigned a decile score from 1 (most disadvantaged 10% of suburbs) to 10 (least disadvantaged 10% of suburbs), and decile scores were grouped into three categories: low (1–3), medium (4–7) and high (8–10). During recruitment, enrolment of men from medium-to-high socio-economic areas was paused once they comprised 70% of the sample within a given cohort. If vacant places remained 1 week prior to the scheduled commencement of the first session, this restriction was lifted and otherwise eligible participants from medium-to-high socio-economic areas were recontacted.

#### Eligibility

Participants were eligible if they: (1) identified as a man, (2) were aged 18–70 years and (3) reported at least mild depressive symptoms in the previous 2 weeks (represented by a score ≥5 on the validated 9-item Patient Health Questionnaire (PHQ-9) depression screening survey).^36^ While a PHQ-9 score ≥10 (moderate symptoms or above) has greater sensitivity for detecting major depression, we applied the lower threshold given existing diagnostic scales often underestimate men’s experiences of depression^37^ and increases the generalisability of findings. Participants were not excluded if they were taking antidepressant medication or attending therapy, provided they had not commenced a new medication or therapy, or changed their dosage or frequency, within the 4 weeks prior to completing the baseline survey. Participants required a medical clearance from their general practitioner if they were at a higher risk of experiencing an adverse event due to exercise, based on their responses to a screening questionnaire.^38^ Additional eligibility criteria are presented in box 1. Written, informed consent prior to enrolment was required from all participants (see online supplemental material).

Box 1 **Eligibility criteria at time of enrolment for the randomised trial**

- Identify as a man.
- Aged 18–70 years.
- Report at least mild depressive symptoms in the previous 2 weeks (PHQ-9 ≥5).
- Access to an internet connected smartphone, tablet or computer to complete surveys.
- Has not started receiving or changed frequency of psychotherapy in the past 4 weeks.
- Has not started a new medication or changed medication dose in the past 4 weeks.
- Able to speak, read and understand English.
- Willing to be randomly allocated into either study group.
- Able to walk for 1 hour.
- Available to attend 10 fortnightly therapy sessions at the University of Newcastle over 20 weeks and not planning to move out of the area during the study period.
- No significant risk of suicide (determined after psychologist consultation for any men reporting current thoughts of suicide or self-harm in screening).

PHQ-9, 9-item Patient Health Questionnaire

#### Randomisation and allocation

Within each cohort, randomisation occurred after all participants had completed their baseline assessment and had been scheduled for a recurring fortnightly session time. At this stage, the trial coordinator sent a blinded version of the schedule (with no participant names) to a university staff member who was not affiliated with the trial. The external staff member then generated the randomisation sequences using the online Sealed Envelope randomisation tool and allocated the therapy timeslots to either ‘conventional indoor therapy’ or ‘walk-and-talk therapy’ according to this sequence. To ensure each psychologist received an equal share of participants from both groups, but no more than three walk-and-talk sessions on their typical workday, the schedule was stratified by psychologist and blocked in groups of four in a 1:1 ratio. Allocation concealment was maintained throughout enrolment and baseline data collection: eligibility was determined by the research team based on completion of an online screener, after which eligible participants were emailed a baseline survey preceded by the study consent form (see online supplemental material). Because randomisation did not occur until all baseline data had been collected within a cohort, the research team had no access to allocation sequences during this period. After generating the randomisation sequence, the external staff member emailed the completed schedule to the trial coordinator, who informed participants about their condition assignment prior to the first scheduled session.

#### Interventions

In both conditions, participants were invited to attend 10 fortnightly 60-minute individual psychotherapy sessions over 20 weeks. Participants were scheduled to attend one session per fortnight on the same day and time, with the same psychologist and within their allocated condition. If a participant missed a session, they were offered the opportunity to reschedule with the same psychologist within a time slot allocated to their assigned treatment condition, pending psychologist availability. There was no cost to participants for the sessions.

The sessions were delivered by provisionally registered (n=1) or registered (n=12) psychologists. The provisional psychologist received weekly supervision from a senior registered clinical psychologist during the trial. To prevent confounding, all psychologists delivered therapy in both study arms. Members of the research team delivered a half-day training session for the psychologists prior to working with participants, which involved walking the therapy route, contingency planning, strategies for maintaining safety and confidentiality and details on the proposed therapy style.

The sessions involved a non‐directive supportive therapy style using counselling micro‐skills to address important issues identified by the participant.^39^ This approach remained consistent in each study arm and was selected for the current study for several reasons: (1) it could be feasibly delivered in both indoor and outdoor environments (compared with other approaches which require access to worksheets, manuals or other resources), (2) it maintained the focus of the trial on the experimental manipulation (ie, the conventional indoor vs walk-and-talk context), (3) all psychologists in Australia are required to demonstrate competency with this approach during their training and (4) it is effective for treating depression.^39 40^

Depending on which group they were randomised to, participants received therapy in a conventional indoor seated environment (usual care) or an outdoor, walking environment (walk-and-talk) for the duration of the study. In the conventional indoor therapy arm, sessions occurred in private consultation rooms in the Behavioural Sciences (W) Building at the University of Newcastle Callaghan Campus. The space included two sound-proofed consultation rooms, a research office and a private waiting area. The consultation rooms each contained a bookshelf, two large therapy chairs, a coffee table and a window (approximately 1 m × 2 m) with a view of surrounding campus infrastructure (ie, paved walkways and buildings), with some greenery visible (ie, monstera plants and lawn). In contrast, the walk-and-talk therapy sessions began outside the office and then occurred while walking along an approximately 4 km predetermined route on the University campus. Psychologists were advised to follow the same walking route with participants for each session, but were permitted to make minor variations (eg, extending or shortening the route, or sitting at an outdoor bench for part of the session) based on participant capacity and environmental conditions (eg, heat or heavy rain). To minimise any physical risks, the route followed well-maintained walking paths on campus, including both built elements (eg, paved footpaths and near buildings) and non-built elements (ie, a 20-minute bushwalk component in a biodiverse nature reserve through the university wetlands) (see online supplemental figures 1 and 2). Participants in the walk-and-talk group were provided with umbrellas, sunscreen, and/or insect repellent, as needed, to maintain physical safety standards during the sessions.

### Safety processes

#### Contact information and mental health support services

Participants were given details on how to contact the research team by email or phone and were made aware of several mental health support services from the commencement of any involvement in the study (eg, in the participant information statement). All emails to the study email account received an automated reply with contact information for 24-hour mental health support services and a confirmation we would get in touch within 72 hours, to allow for weekends when the account would not be monitored (outside of Monday to Friday from 09:00 to 17:00). Those who did not meet eligibility for the trial received an email notifying them of their ineligibility with details of crisis referral information for seeking help and accessing support (ie, the mental health support services).

#### Mental health monitoring

Exacerbations in psychological distress were monitored and thus identified via: (1) recent suicidal ideation reported during the screening questionnaire or subsequent study assessments, (2) symptom severity category increases as indicated by the 21-item version of the Depression, Anxiety, and Stress Scale (DASS-21) subscale scores (depression, anxiety and stress) between assessment time periods, (3) contact to the chief investigator, or (4) requests for assistance received via the study email. In response to exacerbations in psychological distress by participants, a registered psychologist contacted the participant directly via telephone within a maximum 72 hours to determine the exact nature of the distress and the best course of action to support them (ie, existing pathways to care (eg, advise participant to contact their general practitioner (GP)), 24 hours support information (eg, Life Line https://www.lifeline.org.au/) and available referral options (eg, Men’s Line https://mensline.org.au/) based on the NSW Health Framework for Suicide Risk Assessment and Management with their clinical judgement). All instances of exacerbations in psychological distress during the study were recorded as adverse events in the participant’s file along with the results of the risk assessment undertaken by the psychologist and any action taken in response to this. Clinical supervision with a senior registered clinical psychologist to discuss these decisions was used where required. Ineligible participants (as described in the previous section), as well as participants who dropped out of the trial for any reason were provided with the same access to a study psychologist as an enrolled participant.

#### Walking safety protocol

Several checks to mitigate risks and ensure a safe physical environment for the outdoor walking sessions were implemented. Before the first session, clients were advised to wear appropriate footwear and clothing, and to bring a water bottle. Psychologists provided a safety briefing detailing the planned route, potential hazards and safety precautions. While the eligibility screener confirmed that clients did not have medical conditions, mobility issues or other concerns that might impede their ability to walk safely for 60 min, the client set the walking pace and was encouraged to voice any discomfort or the need for breaks. Prior to each session, psychologists checked weather forecasts and ensured they were prepared for possible weather changes. Conventional indoor sessions were substituted for walking sessions during extreme conditions (eg, thunderstorms). Psychologists and participants checked out with a staff member at the start of each session so that their whereabouts was accounted for. During sessions, psychologists also carried their mobile phones equipped with the university’s security contact number.

#### Unwanted intervention-related events

To monitor safety and potential harms, psychologists completed an adapted version of the Unwanted Events and Adverse Treatment Reactions checklist (Linden, 2013) via the post-session online log. For each session, psychologists recorded whether any intervention-related unwanted events (UEs) occurred, and if so, identified the event class (eg, emergence of new symptoms, deterioration of symptoms), rated its relation to the intervention and rated severity across five levels ranging from mild without consequences to extremely severe requiring hospitalisation or posing a life-threatening risk. A brief open-response description was also recorded for each UE. Severe adverse event would lead to contact from an independent research psychologist. For the current study, we adapted the measure to include additional trial-specific event categories (eg, privacy concern, physical injury) and restrict reporting only to events psychologists considered related or probably related to the intervention.

### Measures

Participants completed three online surveys (baseline, post-intervention at 5 months and 12-month follow-up) via QuestionPro, a secure online survey platform licensed by the University of Newcastle. The baseline survey was completed after all participants were enrolled and prior to their first therapy session. The post-intervention survey was completed approximately 1 week after their last therapy session (which was at 5 months post baseline), and the follow-up survey will be completed at 12 months post baseline. Participants will receive an email containing a personalised survey link at each time point. To promote retention and maximise completions at post-study and follow-up, participants who have not responded within 1 week will receive a reminder email, followed by a reminder telephone call and text message if needed. Participants who remain unresponsive will be sent an abbreviated survey containing the primary outcome measure only (DASS-21), consistent with an intention-to-treat approach. At the end of each week, survey data will be exported from QuestionPro and imported into a password-protected database stored on a secure University of Newcastle OneDrive server. All identifying information will be replaced with a unique numerical participant code prior to analysis; however, a separate password-protected linkage file will be retained by the research team to enable longitudinal data linkage across time points. Only members of the research team will have access to identifiable data.

#### Primary outcome

##### Psychological distress

Overall psychological distress was assessed using the composite score of the DASS-21 measure.^41^ The DASS-21 is a widely used and extensively validated measure of commonly experienced symptoms associated with depression, anxiety and stress over the previous week. Participants rated each item on a 4-point scale ranging from 0 (did not apply to me) to 3 (applied to me very much). To allow for comparison with the original DASS-42 scale, total scores were calculated and then doubled as recommended, with higher scores indicating worse mental health.

Although men with depressive symptoms are the target sample, the primary outcome was informed by pilot trial data, which showed comparable reductions in depressive symptoms across conditions but a potential signal favouring walk-and-talk therapy on overall psychological distress. Given anxiety and stress commonly co-occur with depression,^42^ the current trial was designed to provide an adequately powered test of this preliminary effect on overall distress, which also has a theoretical basis given the established links between physical activity,^43^ nature exposure^44^ and symptoms of depression, anxiety and stress.^45 46^

### Survey measures

Survey measures including secondary outcomes and other measures are outlined in table 1. Other measures included treatment expectancies and preferences, and therapeutic process indicators.

**Table 1:** Survey measures

Outcome	Measure	Reference	Items	Example item	Anchors	Base-line	5-month	12-month
Primary outcome								
Overall psychological distress	Depression, Anxiety and Stress Scale (DASS-21)	^41^	21	See below	4-point scale: Did not apply to me (0) to applied to me very much (3)	X	X	X
Secondary outcomes								
Depression	Depression subscale (DASS-21)	^41^	7	I couldn't seem to experience any positive feeling at all	4-point scale: Did not apply to me (0) to applied to me very much (3)	X	X	X
Anxiety	Anxiety subscale (DASS-21)	^41^	7	I was aware of dryness of my mouth	4-point scale: Did not apply to me (0) to applied to me very much (3)	X	X	X
Stress	Stress subscale (DASS-21)	^41^	7	I found it hard to wind down	4-point scale: Did not apply to me (0) to applied to me very much (3)	X	X	X
Male-type depression symptoms	Male Depression Risk Scale (MDRS-7)	^54^	7	I overreacted to situations with aggressive behaviour	5-point scale: None of the time (0) to all the time (4)	X	X	X
Mental well-being	Warwick-Edinburgh Mental Well-being Scale – Short Version (SWEMWBS)	^55^	7	I’ve been thinking clearly	5-point scale: None of the time (1) to all the time (5)	X	X	X
Suicidal ideation	Suicidal Ideation Attributes Scale (SIDAS)	^56^	5	In the past month, how often have you had thoughts about suicide?	10-point scale: Anchors vary by item	X	X	X
Quality of life	Short Form-12 survey (SF-12)	^57^	12	In general, would you say your health is:	Varied	X	X	X
Physical activity	International Physical Activity Questionnaire (IPAQ) Short Form	^58^	6	During the last 7 days, on how many days did you walk for at least 10 min at a time?	Open response	X	X	
Sleep quality	Brief Version of the Pittsburgh Sleep Quality Index (B-PSQI)	^59^	6	During the past month, when have you usually gone to bed at night?	Varied	X	X	
Mental restoration	Restoration Outcome Scale (ROS-6)	^60^	6	When I was participating in my therapy sessions, my concentration and alertness clearly increased	7-point scale: Not at all (1) to totally (7)		X	
Engagement in valued activities	Behavioural Activation for Depression Scale Short Form (BADS-SF)	^61^	9	I was an active person and accomplished the goals I set out to do	8-point scale: Not at all (0) to completely (7)	X	X	
Emotional control	Conformity to Masculine Norms-46 (CMNI-46): Emotional Control subscale	^62^	6	I hate it when people ask me to talk about my feelings	4-point scale: Strongly disagree (1) to strongly agree (4)	X		
Nature connection	Connection with Nature Instrument (CN-12)	^63^	12	I feel right at home when I am in nature	7-point scale: Strongly disagree (1) to strongly agree (7)	X	X	
Nature contact	Nature Contact	^64^	1	Over the past month, approximately how many hours per week on average would you consider yourself to have interacted with nature? For example, walking outside, biking, gardening, playing games/sports, camping, fishing, reading outside, yard work, hanging out in a park, etc	Open response	X	X	
Other measures								
Therapy expectancies and preferences	Treatment Acceptability Preferences Scale (TAPS): Expectancies	^65^	8	How effective do you think this approach would be for improving your mood?	5-point scale: Not at all (0) to very much (4)	X		
	Treatment Acceptability Preferences Scale (TAPS): Preference		1	Do you have a preference for one of the two approaches to therapy?	No; yes - outdoor walk-and-talk; yes - conventional indoor	X		
Therapeutic process indicators	Individual Therapy Process Questionnaire (ITPQ): Global Alliance and Patient Fear subscales	^66^	5	Today, I felt comfortable in the relationship with the therapist (Global Alliance) Today, it was difficult for me to talk openly with the therapist (Patient Fear)	5-point scale: Does not apply (0) to fully applies (4)	*		

* Measure completed via texted survey after participants’ first, middle and final session.

### Physical activity

Physical activity was measured using YAMAX SW200 pedometers. Participants were given a pedometer and log sheet to record their daily step count over 7 days. For the baseline measurement, participants were express mailed the pedometer and log sheet approximately 9 days prior to their first session (allowing 2 days for postage and 7 days for logging) and were asked to return them at their first session. Similarly, for the post-intervention measurement, participants were mailed the pedometer, log sheet and a paid reply package prior to their last session, and were asked to mail them once they have completed/recorded their step count over 7 days, or return it at their last session.

### Attendance, fidelity and adherence

Psychologists completed a post-session online log for every attended session, where they recorded whether the client attended (attendance – yes/no) and whether they were able to conduct the session in the intended setting (fidelity – yes/no). In addition, using a scale created for the purpose of this study, psychologists reported how well they aligned with a non-directive supportive therapy approach during the session (adherence – scale where 0=no adherence to 10=complete adherence).

### Costs

The costs of delivering walk-and-talk (outdoor group) and usual care (indoor group), including psychologist, other staff time and space requirements, were recorded. Health service use data (eg, GP visits, emergency presentations, hospital admission, etc, during the last 5 months) were collected using client self-report. Costs related to service use were derived from publicly available sources including Medicare Benefits Schedule, and Pharmaceutical Benefits Scheme item codes and Diagnosis-Related Groups (for hospitalisation data).

### Demographics

To examine the representativeness of the sample, participants provided demographic information including age, self-reported employment status, education level, country of birth, ethnicity, primary language, marital status and whether they were Aboriginal and/or Torres Strait Islander. Participants also reported how they heard about the study (eg, social media ad, radio interview, flyer).

### Sample size

A sample size of 56 per group at 5 months would provide 80% power to detect a between group difference (in change score) of 8 units in overall psychological distress (DASS-21, α=0.05, SD change=15 units). This aligns with our pilot effect, which also represented an 18% reduction in baseline symptoms.^33^ While this is at the lower threshold of clinical significance, we expect this difference will be evident above any improvements reported by the conventional indoor therapy condition. In another trial targeting men with low mood, a 21% attrition was experienced over 6 months.^47^ Based on this, we conservatively estimated a 25% attrition rate at 5 months (the primary endpoint). To ensure complete data from 56 men per group at 5 months, we aimed to randomise at least 150 men into the trial (ie, 75 per group).

### Patient and public involvement

A lived experience representative and end-user (EW) was involved in this study as an associate investigator from the grant application stage and is a coauthor on this protocol. His involvement included input into study design, including selection of outcome measures based on lived experience and relevance to participants. EW also reviewed and provided feedback on participant-facing materials, including recruitment materials, participant information statements and written communication templates. EW will continue to be involved throughout the trial, including interpretation of findings and contribution to dissemination outputs, and will be included as a coauthor on resulting trial publications. In addition, the study design was informed by feedback from men with lived experience who participated in a preregistered pilot study.^33^ Based on feedback regarding burden and engagement, the intervention was refined from weekly to fortnightly sessions and extended in duration to better align with participant preferences.

The trial was co-designed with health service stakeholders, including a clinical psychologist representing Hunter New England Mental Health, who contributed to the intervention schedule, psychologist recruitment and harm monitoring procedures. Trial governance is provided by an Executive Committee comprising all investigators and the postdoctoral researcher, who met regularly to monitor progress. An End-User Committee (chief investigator, postdoctoral researcher, lived experience representative, external clinical psychologist) will meet biannually to provide independent oversight, review progress against milestones and contribute to interpretation of findings and coauthorship of trial publications. A formal Data Monitoring Committee was not established due to the low-risk nature of both interventions (standard individual psychological counselling differing only in setting), the absence of pharmacological risk and the short duration of each phase. Interim data were not made available to investigators or external parties during the trial period to preserve outcome assessment integrity.

### Statistical methods

#### Primary analysis plan

Linear mixed models will examine the primary and secondary outcomes for the impact of group, time (categorical) and the group-by-time interaction. This approach ensures outcomes for participants lost to follow-up are modelled in the analyses, consistent with an intention-to-treat approach. To adjust for potential baseline differences, age, socio-economic status, baseline physical health (Short Form-12 survey (SF-12) physical subscale) and baseline depression treatment (ie, concurrent use of antidepressant medication and/or external psychotherapy) will be examined to determine whether they contribute significantly to the models. If a covariate is significant, a term will be added to the model to adjust for the effects and two-way interactions with time and treatment will also be examined. If these interactions are significant, they will also be adjusted for. For the primary outcome, we will conduct a sensitivity analysis to explore the impact of adjusting for participants’ prerandomisation treatment expectancies (for the arm they were subsequently randomised to) on the group-by-time intervention effect. A post-hoc sensitivity analysis will also be conducted to account for therapist-level clustering by modelling therapist as a random intercept term, enabling direct comparison of results with and without this effect.

As the analyses of secondary outcomes are intended to complement the primary outcome data and provide preliminary insights for future hypothesis testing, no multiplicity adjustments will be conducted. Given the exploratory nature of these secondary analyses, p values <0.05 will be interpreted as suggestive, rather than significant effects for these outcomes. Effect sizes will be calculated using Cohen’s *d* and interpreted as small (*d*=0.2), medium (*d*=0.5) or large (*d*=0.8).^48^

#### Moderator analysis plan

Potential moderators of intervention effects for the study primary and secondary outcomes will be examined using interaction tests in the linear mixed models. Subgroup analyses will be conducted for preregistered moderator variables with significance of the group-by-moderator interaction where p≤0.10: *age* (dichotomised at the median), *baseline psychological distress* (DASS-21, normal-to-mild (<41) vs moderate-to-extremely severe (41+)), *baseline nature connection* (Connection with Nature Instrument (CN-12) score, weak (<5) vs moderate-to-strong (5+)), *baseline emotional control* (Conformity to Masculine Norms-46 (CMNI-46) emotional control subscale, greater restraint (<2) vs lower restraint (2+)) and *baseline physical activity level* (International Physical Activity Questionnaire (IPAQ) short form, inactive vs minimally active/high active).

#### Cost-effectiveness analysis plan

A trial-based cost-utility analysis will be undertaken from the perspective of the health service over a 12-month period. Health utility of patients over the duration of the study will be derived from their SF-12 scores, and quality-adjusted life-years gained will be calculated. Costs of the intervention and health service use will be compared across the two groups and an incremental cost-effectiveness ratio (showing differences in costs and quality-adjusted life years gained) will be calculated. Sensitivity analyses, including probabilistic analyses will be used to assess the robustness of results to uncertainty in estimates of costs and health outcomes.

## Ethics and dissemination

This study has been approved by the University of Newcastle Human Research Ethics Committee (H-2024-0090). The investigators and staff named on the ethics approval will analyse the data and write the trial manuscript. We intend to publish results in an open access peer-reviewed journal as soon as practicable after study completion. Findings will be disseminated through community-facing presentations and presentations at national and international academic conferences. In addition, policy briefs will be prepared and shared with relevant stakeholders to support translation of findings into practice.

## Discussion

Given over 100 million men worldwide are affected by depression,^49^ and at least one in eight Australian men experience it annually,^5^ yet less than 40% attend therapy with nearly half discontinuing prematurely,^16^ there is an urgent need to evaluate novel approaches to engage and assist men experiencing poor mental health. Walk-and-talk therapy, which integrates physical activity with psychological support in outdoor environments, has emerged as a promising approach to address these challenges.^50 51^ This protocol outlines our study that aims to compare the effectiveness of walk-and-talk therapy to conventional indoor therapy on overall psychological distress in men with depressive symptoms. Despite the clear need for targeted therapy approaches for men, this study will also be one of the only clinical trials specifically targeting men with depression in the past decade.^24^

Strengths of this study protocol include a randomised design with an active intervention control group (conventional indoor therapy), a preregistered, detailed statistical analysis plan that will follow the intention-to-treat principle and the inclusion of a 12-month follow-up to measure the longer-term treatment outcomes. In addition to measuring overall psychological distress (depression, anxiety and stress), a comprehensive range of secondary outcomes will also be assessed to capture other psychological (male-type depression symptoms, mental well-being, suicidal ideation, quality of life), behavioural (physical activity, sleep, nature contact), social (nature connection, emotional control, engagement in valued activities, mental restoration) and economic (costs) impacts of the programme. By successfully completing this trial, insights into the efficacy of an innovative psychotherapy approach for men will be generated through various analytical methods (eg, mediation and moderation analyses). Lastly, recruitment efforts will focus on men residing in areas identified as socio-economically disadvantaged to ensure a diverse sample. Limitations of the current study include reliance on psychologist self-report to assess adherence to the therapy modality, as sessions are not independently coded. This approach was necessary due to the practical challenges of recording sessions in outdoor settings; however, it may introduce bias into the fidelity assessment. Furthermore, data relating to therapist favourability to a particular therapy setting were not collected, which may represent an additional source of bias; future trials building on this work would benefit from collecting therapist allegiance data to enable assessment of its potential influence in sensitivity analyses. The power calculation did not account for therapist-level clustering; the extent to which this affects statistical power will depend on the magnitude of the therapist-level intraclass correlation coefficient, which will be reported alongside the trial outcomes. In addition, participants self-select into a trial conducted at a university campus, which may limit generalisability to other clinical populations and health service settings. Individuals who are willing to participate in such trials may be more motivated or more receptive to novel therapeutic approaches than the general population. As such, further research will be needed to evaluate this intervention when implemented within routine care contexts.

This study will provide new evidence on the effectiveness, safety and cost-utility of two alternative therapeutic approaches, informing whether walk-and-talk therapy should be integrated into routine health practice. Should walk-and-talk therapy demonstrate comparable or superior outcomes to conventional indoor therapy, findings may support its adoption as an alternative therapeutic option within existing psychological services and/or inform the development of practice guidelines. In particular, offering therapy in outdoor settings may improve engagement among men who are less likely to access or remain in traditional clinic-based psychotherapy. Although our study focuses on efficacy rather than implementation, we have collaborated closely with end-users, including registered psychologists, men with lived experience (EW) and health service managers. This collaboration ensures that our study addresses important considerations for men with depression and tests an intervention with realistic potential for health service implementation. Lastly, regarding cost-utility, economic evaluations conducted alongside clinical trials are increasingly recommended to inform health policy and resource allocation in mental health services.^52^ While there is currently limited evidence regarding nature-based therapeutic interventions’ economic value relative to traditional psychotherapy, there is some evidence for its potential cost-effectiveness for people with mild to moderate common mental health problems but significant further research is required.^53^

## Supplementary material

10.1136/bmjopen-2026-122564 online supplemental file 1

10.1136/bmjopen-2026-122564 online supplemental figure 1

10.1136/bmjopen-2026-122564 online supplemental figure 2
