# Supplementary material for: Walk-and-talk versus conventional psychotherapy for men with depressive symptoms: study protocol of a randomised controlled trial
Source: BMJ Open. 2026 Aug 26;16(8):e122564. doi: 10.1136/bmjopen-2026-122564 (PMC13536115; doi:10.1136/bmjopen-2026-122564)
Supplement: Supplementary file 1 [file bmjopen-16-8-s001.pdf]

Walk-and-Talk vs Indoor Therapy for Men with Low Mood

Participant Consent Form

- I agree to participate in the above research project and give my consent freely.
- I understand that the project will be conducted as described in the Participant Information Statement, a copy of which I have read and retained.
- I understand that I can withdraw from the project as explained in the Participant Information Statement, and I do not have to give any reason for my decision to withdraw.

I consent to:

- Attending 10 one-hour therapy sessions with a registered or provisionally registered psychologist over 20 weeks at the University of Newcastle, Callaghan Campus (once per fortnight);
- Being randomly allocated to complete the therapy session either: 1) sitting indoors or 2) walking outdoors.
- Completing an online survey at three timepoints: pre-study (baseline), a post-study (5-months post baseline), and a follow-up (12-months post baseline).
- Wearing a physical activity monitoring device for seven (7) days at three timepoints (noted above).

I understand that my personal information will remain confidential to the researchers, except as required by law.

I have had the opportunity to have questions answered to my satisfaction.

Participant Details

First name: ______________________________

Last name: ______________________________

Email address: ______________________________

Receiving Research Results

I would like the research team to email me a summary of the research results once available.

☐ Yes

☐ No

Please sign here: ______________________________
