# Supplementary material for: Walk-and-talk versus conventional psychotherapy for men with depressive symptoms: study protocol of a randomised controlled trial
Source: BMJ Open. 2026 Aug 26;16(8):e122564. doi: 10.1136/bmjopen-2026-122564 (PMC13536115; doi:10.1136/bmjopen-2026-122564)

Supplementary Figure 1. Built elements of walk-and-talk route.

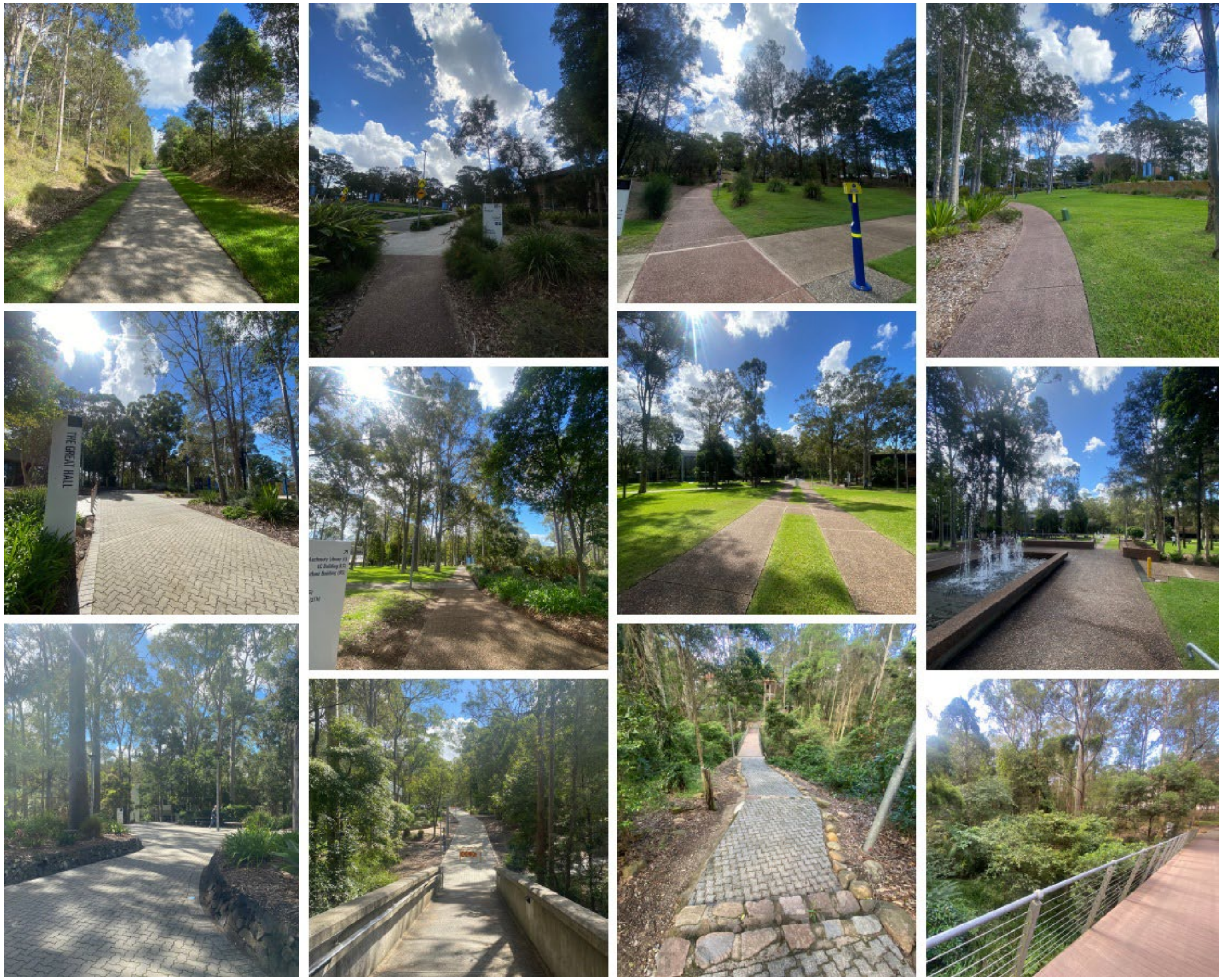

Supplement: Supplementary file 2 [file bmjopen-16-8-s002.pdf]
