# Supplementary material for: Walk-and-talk versus conventional psychotherapy for men with depressive symptoms: study protocol of a randomised controlled trial
Source: BMJ Open. 2026 Aug 26;16(8):e122564. doi: 10.1136/bmjopen-2026-122564 (PMC13536115; doi:10.1136/bmjopen-2026-122564)
Supplement: Supplementary file 4 [file bmjopen-2026-122564.reviewer_comments.pdf]

PEER REVIEW HISTORY

BMJ Open publishes all reviews undertaken for accepted manuscripts. Reviewers are asked to complete a checklist review form and are provided with free text boxes to elaborate on their assessment. These free text comments are reproduced below.

ARTICLE DETAILS

Title (Provisional)

Walk-and-talk vs conventional psychotherapy for men with depressive symptoms: Study protocol of a randomised controlled trial

Authors

Regan, Casey Phyllis; Dickmeyer, Andrea; O'Hara, Robert; Halpin, Sean Anthony; Smith, Jordan James; Morgan, Philip; Drew, Ryan J; McCreanor, Victoria; Valkenborghs, Sarah Ruth; Waters, Elliot; Seidler, Zac; Young, Myles D

VERSION 1 - REVIEW

**Reviewer**	**1**
**Name**	**Guthrie, Elspeth**
**Affiliation**	**University of Leeds Leeds Institute of Health Sciences, Psychological and Social Medicine**
**Date**	**05-Jun-2026**
**COI**	**None**

This manuscript describes a protocol for a randomised controlled trial of walking psychological treatment versus usual office based psychological treatment for male identifying university students.

It is well recognised that it is difficult to recruit men to psychological treatment studies and for men to engage in therapy so this is an intervention that is worth investigating and there is a clear rationale for testing it against a more conventional delivery of therapy.

The therapy itself also seems suitable for people with mild to moderate psychological distress and the team have embedded people with lived experience throughout the development of the project.

Safety procedures and reporting seem adequate and well considered.

The manuscript is clear and well written. The researchers have already conducted a feasibility study to test recruitment, delivery of treatments, measures etc.

The researchers have accounted for attrition in their power calculation.

However, I have some concerns about the power. The researchers do not appear to have accounted for therapist variability in the trial. Each therapist acts in effect as their own cluster. There are 13 therapists so this is likely to impact the power. As they have actually completed the intervention and the sample size cannot be changed, I would strongly suggest they include therapist variability in their SAP .It is likely to have a substantial effect on the power and I think it is unlikely that the study will be sufficiently powered. The team should have some data from the feasibility regarding therapist variability. If not there are several guides and estimates that have been written to help trialists estimate therapist variability.

Therapists have delivered both forms of therapy. Although this reduces one form of bias, it introduces another potential bias in that therapists may favour one approach over the other. There is of course potential further bias, which the team acknowledge, in that therapists have been asked to rate their own adherence and delivery of the therapy.

There is good evidence from meta-analyses that bone fida therapies delivered well show very little difference when tested head to head against each other.

I was surprised that there was not a DMEC. DMECs do not just oversee risk but they also can provide valuable input regarding the statistical analysis plan (SAP) etc

Overall this will be a valuable addition to the literature, even if it turns out it is not powered sufficiently, as it could lead to further funding for a larger multi site study.

VERSION 1 - AUTHOR RESPONSE

#	Source	Comment	Author Response	Revision Made (Line Numbers)
5–9, 12, 15	Reviewer 1	The manuscript describes a protocol for a randomised controlled trial of walking psychological treatment versus usual office based psychological treatment for male identifying university students. It is well recognised that it is difficult to recruit men to psychological treatment studies and for men to engage in therapy so this is an intervention that is worth investigating and there is a clear rationale for testing it against a more conventional delivery of therapy.<br><br>The therapy itself also seems suitable for people with mild to moderate psychological	We thank the reviewer for these positive observations and their careful and constructive review of our manuscript.	No change required.

#	Source	Comment	Author Response	Revision Made (Line Numbers)
		distress and the team have embedded people with lived experience throughout the development of the project.<br><br>Safety procedures and reporting seem adequate and well considered.<br><br>The manuscript is clear and well written. The researchers have already conducted a feasibility study to test recruitment, delivery of treatments, measures etc.<br><br>The researchers have accounted for attrition in their power calculation.<br><br>There is further potential bias, which the team acknowledge, in that therapists have been asked to rate their own adherence and delivery of the therapy.<br><br>Overall this will be a valuable addition to the literature, even if it turns out it is not powered sufficiently, as it could lead to further funding for a larger multi site study.		
10	Reviewer 1	The researchers do not appear to have accounted for therapist variability in the trial. Each therapist acts in effect as their own cluster. There are 13 therapists so this is likely to impact the power. As they have actually completed the intervention and the sample size cannot be changed, I would strongly suggest they include therapist variability in their SAP. It is likely to have a substantial effect on the power and I think it is unlikely that the study will be sufficiently powered. The team should have some data from the	We acknowledge that therapist-level clustering was not sufficiently addressed in the original protocol. We have updated both the protocol and trial registry to include a post-hoc sensitivity analysis which will account for clustering by modelling therapist as a random intercept term. We have also added a sentence to the limitations to acknowledge this. Given published intraclass correlation	**Line 423-426**: The sentence **“**A post-hoc sensitivity analysis will also be conducted to account for therapist-level clustering by modelling therapist as a random intercept term, enabling direct comparison of results with and without this effect” was added to the methods section.<br><br>The above was also applied to the ANZCTR trial registry

#	Source	Comment	Author Response	Revision Made (Line Numbers)
		feasibility regarding therapist variability. If not there are several guides and estimates that have been written to help trialists estimate therapist variability.	coefficient (ICC) data for non-directive supportive therapy approaches remain limited, we will also report therapist-level ICCs to inform the design of future trials.	**Line 493-496**: The sentence "The power calculation did not account for therapist-level clustering; the extent to which this affects statistical power will depend on the magnitude of the therapist-level intraclass correlation coefficient, which will be reported alongside the trial outcomes" was added to the limitations discussion.
11	Reviewer 1	Therapists have delivered both forms of therapy. Although this reduces one form of bias, it introduces another potential bias in that therapists may favour one approach over the other.	We agree that therapist preference for a particular therapy setting may represent a source of bias, and that data relating to this was not collected. This is acknowledged as a limitation of the current trial, and future studies building on this work would benefit from collecting therapist allegiance data to enable exploration of its potential influence in sensitivity analyses.	**Line 490-493**: The sentence "Furthermore, data relating to therapist favourability to a particular therapy setting were not collected, which may represent an additional source of bias; future trials building on this work would benefit from collecting therapist allegiance data to enable assessment of its potential influence in sensitivity analyses" was added to limitations.
13	Reviewer 1	There is good evidence from meta-analyses that bona fide therapies delivered well show very little difference when tested head to head against each other.	We agree, though also note our trial is somewhat unique in that it compares the same therapeutic approach delivered in two different settings, rather than testing two different therapies head-to-head. Nonetheless, we acknowledge this is an important	No change required.

#	Source	Comment	Author Response	Revision Made (Line Numbers)
			consideration, and it is a key reason we have included moderation analyses to examine whether particular subgroups of participants may derive greater benefit from one condition over the other.	
14	Reviewer 1	I was surprised that there was not a DMEC. DMECs do not just oversee risk but they can also provide valuable input regarding the statistical analysis plan etc.	We take the reviewers point and see how this would have been valuable. We will consider incorporating a formal DMEC in future trials building on this work.	No change required.

VERSION 2 - REVIEW

Reviewer 1

Name Guthrie, Elspeth

Affiliation University of Leeds Leeds Institute of Health Sciences, Psychological and Social Medicine

Date 28-Jul-2026

COI

The authors have addressed my concerns and amended the manuscript
